# Deutsche Apothekerausbildung im internationalen Vergleich

**DOI:** 10.1007/s00103-025-04039-z

**Published:** 2025-04-01

**Authors:** Anita Elaine Weidmann, Freyja Jónsdóttir

**Affiliations:** 1 Fakultät für Chemie und Pharmazie, Abteilung Klinische Pharmazie, Universität Innsbruck, Innrain 80/82, 6020 Innsbruck, Österreich; 2 Faculty of Pharmaceutical Sciences, University of Iceland, Reykjavik, Island; 3 Pharmacy Services, Landspitali – The National University Hospital of Iceland, Reykjavik, Island

**Keywords:** Pharmazie, Apotheker, Pharmaziestudium, Qualifikation, Pharmacy, Pharmacist, Education, Qualification

## Abstract

Während das Pharmaziestudium in Deutschland mit einem Staatsexamen abgeschlossen wird, gibt es international 3 Universitätsbildungssysteme mit den Abschlüssen Master of Science (MSc), Master of Pharmacy (MPharm) und Doctor of Pharmacy (PharmD). Die Inhalte des deutschen Pharmaziestudiums und Staatsexamens werden in der Approbationsordnung für Apotheker (AAppO) festgelegt und legen den Fokus auf pharmazeutische Wissenschaften. Der vom Bologna-Prozess geprägte MSc ist ein akademischer Abschluss, der sich auf fortgeschrittene Studien und Forschung in einem bestimmten Bereich der pharmazeutischen Wissenschaften konzentriert. Der MPharm- und der PharmD-Abschluss, die sich überwiegend im angloamerikanischen Raum etabliert haben, beinhalten eine vorwiegend klinische Ausbildung, um Apotheker auf komplexe Aufgaben im interprofessionellen Team vorzubereiten. Neue Technologien, wissenschaftlicher Fortschritt, aber auch globale Herausforderungen üben einen wichtigen Einfluss auf die Inhalte des Pharmaziestudiums aus, die in sehr unterschiedlicher Geschwindigkeit ihre Berücksichtigung finden. Während in vielen Ländern die fachspezifischen Inhalte und die Erreichung einheitlich festgelegter Kompetenzen durch Akkreditierungsgremien sichergestellt werden, lassen die Studieninhalte in Deutschland keinen größeren Spielraum zur Ausgestaltung zu, was unter anderem durch einen engen Rechtsrahmen und bildungspolitische Fragmentierung bedingt ist. Obgleich das Pharmaziestudium ein hoch qualifizierender Prozess ist, besteht in Europa ein dringender Bedarf, sich um eine stärkere Anpassung der Curricula zu bemühen, um die Erwartungen eines modernen Rollenprofils zu erfüllen. In dieser narrativen Übersichtsarbeit werden die unterschiedlichen Universitätsbildungssysteme und die treibenden Faktoren für die Weiterentwicklung der Pharmaziecurricula beleuchtet.

## Abstract (en)

While a pharmacy degree in Germany uses a state examination system, they are divided into three university education systems worldwide: Master of Science (MSc), Master of Pharmacy (MPharm), and Doctor of Pharmacy (PharmD). The educational content of the German pharmacy degree and state examination is dictated by the “Approbationsordnung für Apotheker” (AAppO) and focuses on pharmaceutical sciences. Shaped by the Bologna Process, the MSc is an academic degree that focuses on advanced studies and research in a specific area of pharmaceutical sciences or related fields. The MPharm and PharmD degrees, which have become established in the Anglo-American region, offer primarily clinical training to prepare pharmacists for complex tasks in an interdisciplinary team. New technologies, scientific progress, and global challenges have an important influence on the content of pharmacist education, which is taken on board at very different speeds. While in many countries the subject-specific content and the achievement of uniformly defined competencies are ensured by accreditation bodies, the course content in countries like Germany does not allow any greater scope for redesign, which is due to rigid legal frameworks and educational policy fragmentation. Although the pharmacy degree is a highly qualifying process, there is an urgent need for European curricula to be more in line with the expectations of a modern pharmacist role profile. This narrative review examines the different university education systems and the driving factors for the further development of the pharmaceutical curriculum.

## Universitätsbildungssysteme im internationalen Vergleich

### Internationale Abschlüsse und Bologna-Prozess in Europa

Weltweit lassen sich beim Pharmaziestudium 3 Universitätsbildungssysteme mit den folgenden Abschlüssen unterscheiden: „Master of Science“ (MSc), „Master of Pharmacy“ (MPharm) und „Doctor of Pharmacy“ (PharmD). In Europa ist das dominante System des Bachelor/Master of Science (BSc/MSc) sehr stark durch den von 29 europäischen Staaten im Jahr 1999 unterzeichneten Bologna-Prozess geprägt [1]. Die Bologna-Erklärung von 1999 zielte darauf ab, innerhalb von 10 Jahren einen europäischen Hochschulraum (European Higher Education Area, EHEA) zu schaffen, in dem die Mobilität von Studierenden, Lehrenden und Forschenden sowie des wissenschaftlichen Hochschulpersonals im Rahmen qualitätsgesicherter, transparenter und vergleichbarer Studienangebote unter Anerkennung der erbrachten Studienleistung möglich ist [2]. Zur Erreichung der Ziele sollte das Studium durch Etablierung des 2‑stufigen Bachelor‑/Master-Systems für berufsqualifizierende Studienabschlüsse harmonisiert werden. Das „European Credit Transfer System“ (ECTS) zum Leistungsvergleich und eine fortlaufende Qualitätssicherung im Hochschulbereich wurden etabliert. In Deutschland mit seiner naturwissenschaftlichen Ausrichtung sollte eine Beschäftigungsfähigkeit der Absolventen in einem breiten Spektrum des pharmazeutischen Sektors ermöglicht werden [3]. Während sich einige Länder einer wesentlichen strukturellen Änderung des Pharmaziestudiums unterzogen haben, um den hohen Standards und beruflichen Anforderungen gerecht zu werden, ermöglichte es die im Rahmen des Bologna-Prozesses gewährte Flexibilität manchen Ländern, ihre Pharmaziestudiumsmodelle beizubehalten [3]. Hierzu gehören unter anderem das in Deutschland bestehende Modell des Staatsexamens und das Französische „Diplôme d’État de Docteur en Pharmacie“ (Staatsdiplom des Doktors der Pharmazie; [4]). Tab. 1 gibt einen Überblick über die verschiedenen Pharmaziestudiengänge.

**Tab. 1:** Überblick zu Pharmaziestudiengängen im internationalen Vergleich (kein Anspruch auf Vollständigkeit)

	Staatsexamen	Bachelor/Master of Science (BSc/MSc)	Bachelor/Master of Pharmacy (BPharm/MPharm)	Doctor of Pharmacy (PharmD)
Schwerpunkt und Zielsetzung	Berufsqualifizierender Abschluss mit einem Fokus auf pharmazeutische Wissenschaften	Fortgeschrittene Studien und Forschung in einem bestimmten Bereich der pharmazeutischen Wissenschaften oder verwandten Gebieten	Berufsqualifizierender Abschluss, der die Studierenden auf eine Karriere als Apotheker vorbereitet	Umfassende Ausbildung in pharmazeutischen Wissenschaften, klinischer Praxis und Patientenversorgung mit Betonung der Rolle der Apotheker als integrale Mitglieder des Gesundheitsteams
Aufbau des Studiengangs	1–2. Staatsexamen 4 Jahre	BSc 3 Jahre MSc 1–3 Jahre (aufbauend)	BPharm 3 Jahre MPharm 4–5 Jahre (nicht aufbauend)	PharmD 6 Jahre
Praxis unter Supervision	Verpflichtend	Freiwillig oder integriert (Niederlande)	Verpflichtend	N/A
Zulassungsprüfung	3. Staatsexamen	Freiwillig oder integriert (Niederlande)	Apothekerkammerprüfung	Nur eine verpflichtende Zulassungsprüfung
Akkreditierung	Nein	Nein	Ja	Ja
Beruflicher Werdegang	Apothekenpraxis, Offizinapotheken, Krankenhausapotheke, Industrie	Wissenschaft, Industrie, Apothekenpraxis oder in staatlichen Einrichtungen	Praktizierende Apotheker in verschiedenen Bereichen, auch in öffentlichen Apotheken und Krankenhäusern	Direkte Patientenversorgung und Management der Arzneimitteltherapie in öffentlichen Apotheken und Krankenhäusern
Spezialisierte Rollen	Pharmaunternehmen, Zulassungsbehörden, klinische Forschungseinrichtungen oder Beratungsunternehmen im Gesundheitswesen	Pharmaunternehmen, Zulassungsbehörden, klinische Forschungseinrichtungen oder Beratungsunternehmen im Gesundheitswesen	Weiterführende Studiengänge sind erforderlich	Spezialisierte Praxisbereiche wie Onkologie, Pädiatrie, Kardiologie, Infektionskrankheiten und mehr; Führungspositionen in Organisationen des Gesundheitswesens, bei Aufsichtsbehörden und in der Wissenschaft
Länder	Deutschland (Staatsexamen)	Island, Italien, Österreich, Portugal, Polen, Schweiz, Schweden, Spanien, Türkei, Niederlande, Norwegen	Australien, Vereinigtes Königreich, Irland, Griechenland	Kanada, Frankreich (Diplôme d’État de Docteur en Pharmacie), Nordamerika, Nigeria, Pakistan, Vereinigte Arabische Emirate, Saudi-Arabien, Indien, Polen (Warschau), Türkei (Istanbul)

### Staatsexamen der Pharmazie in Deutschland

Das Staatsexamen ist eines der ältesten einheitlichen Berufsqualifikationssysteme in Deutschland, mit dem neben dem Pharmaziestudium auch ein Studium in den Fächern Medizin, Lehramt und Jura abgeschlossen wird. Die Approbationsordnung für Apotheker (AAppO), die auch die Ausbildungs- und Prüfungsordnung enthält, wurde inhaltlich zuletzt 2001 aktualisiert, auch wenn organisatorische Elemente, wie die Einrichtung von digitaler Lehre und die Änderungen der Prüfungsmodalitäten, öfter angepasst wurden (zuletzt 2023; [5]). Das heutige Staatsexamen der Pharmazie unterteilt sich in 3 Studienabschnitte [6]. Das Grundstudium dauert in der Regel 4 Semester und beschäftigt sich mit anorganischer und organischer Chemie, Physik, Analytik, Botanik und Histologie von Pflanzen. Daneben werden Grundlagen in Physiologie, Technologie und Mathematik gelehrt, die Famulatur dauert 8 Wochen. Den Abschluss bildet das erste Staatsexamen. Das folgende Hauptstudium besteht aus 4 Semestern. Hierbei werden vor allem die pharmazeutische und medizinische Chemie, pharmazeutische Biologie, pharmazeutische Technologie und Biopharmazie, Pharmakologie und Toxikologie sowie klinische Pharmazie vertieft. Diese Fächer sind auch die Prüfungsfächer des 2. Staatsexamens, wobei Inhalten der klinischen Pharmazie sowie der modernen pharmazeutischen Biologie inzwischen eine viel größere Bedeutung beigemessen wird. Nach erfolgreichem Bestehen des 2. Staatsexamens folgt ein praktisches Jahr. Die Approbationsordnung schreibt hierbei vor, dass mindestens 6 Monate in einer öffentlichen Apotheke absolviert werden müssen. Die verbleibenden 6 Monate können in der pharmazeutischen Industrie, Krankenhausapotheke oder universitären Forschung verbracht werden. Darauf folgt das 3. Staatsexamen (§ 19 AAppO), das eine mündliche Prüfung zu den Fächern Pharmazeutische Praxis und Spezielle Rechtsgebiete für Apotheker umfasst. Nach erfolgreichem Bestehen kann bei den zuständigen Bezirksregierungen die Approbation beantragt werden [7]. Nach dem Staatsexamen, das als Regelabschluss für die Apothekenpraxis gesehen wird, gehen ca. 77,5 % aller Absolventen in öffentliche Apotheken, ca. 4 % in Krankenhausapotheken und 11,4 % in die Industrie (7,1 % andere; [8]).

### Zielsetzung des Bachelor/Master of Science (BSc/MSc) der Pharmazie

Der MSc ist ein akademischer Abschluss, der sich auf fortgeschrittene Studien und Forschung in einem bestimmten Bereich der pharmazeutischen Wissenschaften oder verwandten Gebieten konzentriert [9]. Er bietet fundiertes Wissen in Bereichen wie Pharmakologie, medizinische Chemie, pharmazeutische Technologie, Pharmakoökonomie oder klinische Forschung und beinhaltet in der Regel eine bedeutende Forschungskomponente, oft in Form einer Bachelor- oder Masterarbeit, die auf eigener Forschung basiert [10]. Absolventen können in Pharmaunternehmen, Zulassungsbehörden, klinischen Forschungseinrichtungen oder Beratungsunternehmen im Gesundheitswesen arbeiten. Studierende können ihr Studium auch mit einer Promotion (PhD) fortsetzen, um Karrieren in der Forschung (Wissenschaft, Industrie oder in staatlichen Einrichtungen) zu verfolgen [11]. Ein Master of Science (MSc) dauert in der Regel 1–3 Jahre und baut auf einem erfolgreich absolvierten Bachelor-of-Science-(BSc‑)Abschluss auf. Studierende, die nach dem Abschluss (MSc) eine Karriere in der Apothekenpraxis anstreben, müssen ein zusätzliches Jahr unter pharmazeutischer Aufsicht in der Apotheke arbeiten und eine Zulassungsprüfung bei der landeseigenen Apothekerkammer absolvieren. In manchen Studiengängen ist das Praktikum auch in das Masterstudium integriert (z. B. Niederlande), was auch bedeutet, dass die Zulassungsprüfung Teil des universitären Abschlusses ist und nicht separat dem Land oder der Standesvertretung unterliegt [12].

### Zielsetzung des Bachelor/Master of Pharmacy (BPharm/MPharm)

Im Gegensatz hierzu richtet sich das Bachelor/Master-of-Pharmacy-(BPharm‑/MPharm‑)Studium, das im angloamerikanischen Raum vorherrscht, ausschließlich an diejenigen, die eine Zulassung als Apotheker anstreben [13]. Dementsprechend sind die Studieninhalte überwiegend praxis- und klinisch orientiert. Während der BPharm in asiatischen Ländern den gesetzlichen Anforderungen für die Zulassung als Apotheker entspricht, haben Australien und europäische Länder, wie im Vereinigten Königreich (UK), in Irland, Polen und Ungarn, das Curriculum zum MPharm weiterentwickelt [14]. Hierbei wird der MPharm als Regelabschluss für die Apothekenpraxis gesehen. Studierende, die dabei nicht erfolgreich sind, schließen mit einem BPharm ab; dieser führt zu keiner ausgewiesenen Qualifikation, sondern befähigt sie lediglich zu der Anrechnung der ECTS auf einen anderen Studiengang.

Diese Curriculumsentwicklung geht seit Mitte des 21. Jahrhunderts mit der Rollenprofilentwicklung von Apothekern einher. Wie in anderen Beiträgen beschrieben, hat sich die Rolle der Apotheker von einem primär auf die Abgabe von Arzneimitteln ausgerichteten Beruf zu einem dynamischeren, patientenzentrierten Beruf entwickelt, der anstrebt, zur Therapieoptimierung des Patienten beizutragen. Dieser Wandel spiegelt den allgemeinen Trend zu integrierter Versorgung, personalisierter Medizin und der zunehmenden Komplexität des Gesundheitsmanagements in der heutigen Zeit wider [15]. Der Ausbau der beruflichen Kompetenzen und Arbeitsbereiche sollte sich natürlich auch im Curriculum des Pharmaziestudiums wiederfinden [16].

Der MPharm-Studiengang legt den Schwerpunkt auf die Integration von theoretischem Wissen und praktischen Fähigkeiten, die für die pharmazeutische Praxis erforderlich sind. Dazu gehört eine umfassende Ausbildung im klinischen Umfeld. Der Lehrplan kombiniert pharmazeutische Wissenschaften, Pharmakologie, medizinische Aspekte der Therapieoptimierung und klinische Pharmazie mit Praktika und Hospitationen und ist in der Regel auf die gesetzlichen Zulassungsanforderungen für die Ausübung der Apothekenpraxis im jeweiligen Land abgestimmt [17]. Auf den Abschluss des MPharm-Studiums (nach 4–5 Jahren) folgt in der Regel eine Phase der Praxis unter pharmazeutischer Supervision für ein Jahr und eine Zulassungsprüfung [18]. Um sicherzugehen, dass die einzelnen Studiengänge die Studierenden ausreichend auf die gesetzlichen Zulassungsanforderungen der Apothekenpraxis vorbereiten, werden Curriculumsinhalte und Prüfungsstandards von der jeweiligen Apothekerkammer (z. B. Australian Pharmacy Council; UK General Pharmaceutical Council) im Abstand von 3 bis 5 Jahren kontinuierlich kontrolliert und akkreditiert [19].

### Zielsetzung des Doctor of Pharmacy (PharmD)

Das in Amerika und Kanada vorrangige PharmD-Studium der Pharmazie (Neulateinisch – *Pharmaciae Doctor*) ist stark auf die klinische Praxis, Pharmakotherapie und die unmittelbare Patientenversorgung ausgerichtet und bereitet Apotheker mit umfangreichen klinischen Praktika und interprofessionellem Studium auf komplexe Aufgaben im interprofessionellen Team vor [20]. Wie beim MPharm-Studiengang zielt der PharmD darauf ab, qualifizierte Pharmazeuten auszubilden, aber die Wege und Schwerpunkte unterscheiden sich erheblich und spiegeln die unterschiedlichen Bildungs- und Berufsziele wider. Der PharmD-Abschluss eröffnet Möglichkeiten für spezialisierte Praxisbereiche wie Onkologie, Pädiatrie, Kardiologie, Infektionskrankheiten und mehr. PharmD-Absolventen können Führungspositionen in Organisationen des Gesundheitswesens, bei Aufsichtsbehörden und in der Wissenschaft einnehmen und durch Lehre und Forschung zur Weiterentwicklung des Apothekerberufs beitragen [21]. Dabei ist darauf zu achten, dass er nicht mit einer Dissertation (PhD) gleichzusetzen ist und Absolventen auch keinen Doktortitel tragen. Der PharmD-Abschluss ist ein rein beruflicher Abschluss, der keine eigenständige Forschungsleistung beinhaltet.

### Fort- und Weiterbildungen

**Abb. 1:**
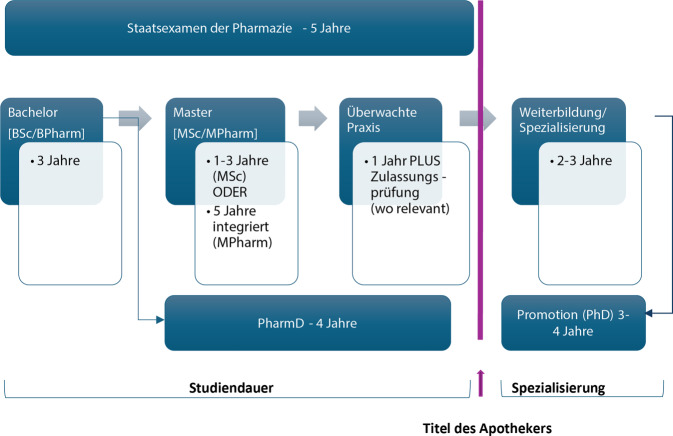
Ablauf der pharmazeutischen Aus- und Weiterbildung im internationalen Vergleich. (Quelle: eigene Abbildung)

Zusätzlich zum Grundstudium sind oft aufbauende Spezialisierungen zur beruflichen Weiterbildung notwendig. Diese variieren je nach Land und Gesundheitssystem in der ausführenden Institution, im Umfang (Fachapotheker 3 Jahre berufsbegleitend oder weiterführender Studiengang [Postgraduate Certificate – PgCert (60 ECTS), Postgraduate Diploma – PgDip (120 ECTS), Master of Science – MSc (180 ECTS)]) und in der verpflichtenden oder nichtverpflichtenden Akkreditierung der weiterführenden Studiumsinhalte durch eine pharmazeutische Kammer [22]. Die häufigsten weiterführenden Studiengänge sind ein MSc in klinischer Pharmazie, ein MSc in Pharmaceutical Sciences und ein MSc in Advanced Practice. Auch anerkannte Weiterbildungen zum Fachapotheker für Klinische Pharmazie (Deutschland, Österreich, Spanien, Portugal, Italien, Kanada, USA, Australien, Südafrika, Island) oder zum verschreibungsbefähigten Apotheker (UK, Irland, Schweden, Finnland, Portugal, USA, Kanada, Neuseeland, Australien) sind vielerorts notwendig, um sich in die Therapie des Patienten einbringen zu dürfen. Somit dauert die Apothekerausbildung, inklusive Weiterbildung bzw. Spezialisierung eines Apothekers, im Schnitt 8–10 Jahre. Der Einstieg in eine Promotion ist nach dem erfolgreichen Abschluss des Staatsexamens oder eines Masters jederzeit möglich (Abb. 1).

Im Folgenden werden Veränderungen in den Lehrinhalten des Pharmaziestudiums beschrieben. Es wird erläutert, wie globale Herausforderungen und nationale Strukturen das Studium beeinflussen, ebenso wie wirtschaftliche und gesundheitsökonomische Faktoren, Qualitätsstandards und Lehrmethoden. Es wird auch auf die Prinzipien der horizontalen und vertikalen Integration im Pharmaziestudium sowie bei Praktika eingegangen.

## Pharmaziecurricula im Wandel

Pharmaziecurricula orientieren sich inhaltlich in erster Linie an den Fortschritten der pharmazeutischen Wissenschaft und Industrie sowie an den Bedarfen des jeweiligen Gesundheitssystems [23]. Hierbei sind wirtschaftliche und gesundheitspolitische Faktoren genauso wichtig wie Kompetenzrichtlinien und Qualitätsstandards der jeweiligen professionellen Organisationen [24]. In Deutschland ist die Pharmazie aus der historisch sehr erfolgreichen Chemieindustrie im Land hervorgegangen und daher in den naturwissenschaftlichen Fakultäten angesiedelt (nur an der Universität in Leipzig ist Pharmazie Teil der medizinischen Fakultät). Daraus resultiert auch die Fokussierung auf die pharmazeutische Chemie [25].

### Einfluss von globalen Herausforderungen und nationalen Strukturen auf das Pharmaziestudium

**Abb. 2:**
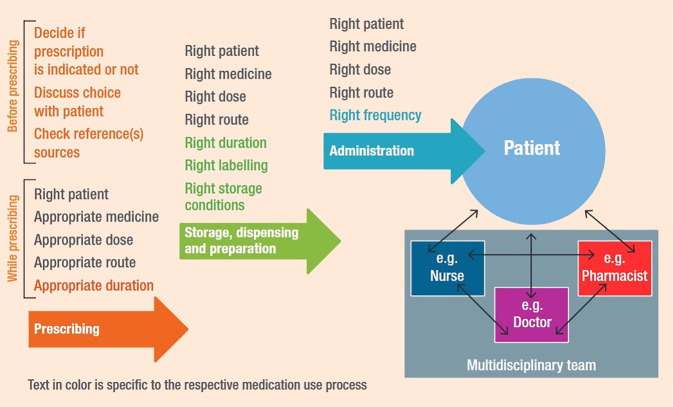
Interprofessionelle Zusammenarbeit im Medikationsprozess. Aus [65]. Lizenz „Attribution-NonCommercial-ShareAlike 3.0 IGO“ (CC BY-NC-SA 3.0 IGO; [66])

In Zeiten von steigenden Bevölkerungszahlen und zunehmendem Durchschnittsalter, chronischen Erkrankungen und Polymedikation sowie Fachkräftemangel und weltweiten Lieferengpässen von Medikamenten wird mit Nachdruck eine effektive Lösung gesucht, um die Kontinuität des Versorgungsstandards zu halten [26]. Hierbei kommt es oft, aufgrund des Ärztemangels, zu einer Erweiterung von Rollen innerhalb der einzelnen Gesundheitsberufe [27, 28]. Weltweit hat sich allerdings der Tätigkeitsbereich der Apotheker im Gesundheitswesen am stärksten weiterentwickelt [29]. Apotheker sind zunehmend an Medikationsanalysen für Patienten mit chronischen Erkrankungen beteiligt, wobei pharmazeutische Dienstleistungen dazu beitragen, die Angemessenheit, korrekte Einnahme, Wirksamkeit und Sicherheit der Medikation zu gewährleisten (USA, Australien, Kanada, UK; [30]). Impfstoffverabreichung, Präventivmedizin, Adhärenzförderung und Patientenschulungen gehören ebenfalls zu den wichtigen Tätigkeitsbereichen, wobei sich Apotheker in vielen Ländern als essenzieller und fest integrierter Bestandteil des interprofessionellen Teams etabliert haben (Abb. 2). Zu diesen erweiterten Tätigkeiten gehört nicht selten eine (un)eingeschränkte Verschreibungsbefugnis von Medikamenten (USA, Kanada, UK; [31]). Diese Erweiterung des Rollenprofils schließt essenzielle Aufgaben wie Herstellung, Logistik und Management mit ein, wie die COVID-19-Pandemie gezeigt hat [32].

Aufbauend auf der vom Europäischen Rat 2006 veröffentlichten Empfehlung „Medication Safety—A specific strategy to promote patient safety“ (Rec(2006)7) hat die Weltgesundheitsorganisation (WHO) eine globale Zielsetzung herausgegeben, um medikationsbezogene Fehler weltweit bis 2030 um 50 % zu senken [33, 34]. Hierbei spielen öffentliche und Krankenhausapotheker eine zentrale Rolle, da sie direkt in den Medikationsprozess und teilweise auch in das interdisziplinäre Team eingebunden sind. Sie verfügen über die notwendigen Kenntnisse und Fähigkeiten, um arzneimittelbezogene Probleme zu identifizieren, zu vermeiden und notwendige Beratungen und Schulungen durchzuführen [35, 36]. Pharmaziecurricula haben sich in vielen Ländern demnach stark an den sich wandelnden Anforderungen und Strukturen des jeweiligen Gesundheitssystems orientiert. In Ländern mit integrierten Gesundheitssystemen, wie dem National Health Service (NHS) des Vereinigten Königreichs, in denen Apotheker immer mehr eine Schlüsselrolle in multidisziplinären Gesundheitsteams spielen, haben sich die Curricula immer stärker auf klinische und interprofessionelle Kompetenzen konzentriert [37]. Daraus folgte 1997 die Weiterentwicklung des BPharm-Curriculums zum MPharm-Studium, um die Studierenden auf ihre zunehmend komplexen Rollen im Gesundheitswesen vorzubereiten [38]. Diese werden durch Kompetenzrahmen untermauert, die jedes Stadium des beruflichen Werdeganges unterstützen und die kontinuierlich verpflichtende Weiterbildung widerspiegeln, von der die jährliche Berufsberechtigung eines Apothekers abhängt [39].

In Ländern mit stärker fragmentierten Gesundheitssystemen, wie Deutschland und Österreich, wird dagegen aufgrund der Nähe zur pharmazeutischen Industrie ein breiter aufgestelltes Pharmaziestudium angeboten (Staatsexamen/MSc), das sich hauptsächlich auf die pharmazeutischen Wissenschaften konzentriert [40]. Die Anpassung des Pharmaziestudiums an das Berufsbild des Apothekers in Deutschland geht nur langsam voran. Hierfür sind vor allem der enge Rechtsrahmen, die bildungspolitische Fragmentierung (Bildung ist Ländersache), unterschiedliche Prioritäten der Bundesländer, hohe Kosten und mangelnde Flexibilität in den universitären Strukturen verantwortlich [41]. Erst im Jahr 2022 sprach sich die Mitgliederversammlung der Bundesapothekerkammer (BAK) dafür aus, die Inhalte des Pharmaziestudiums zu modernisieren [42].

Eine im Jahr 2014 veröffentlichte Studie zeigte auf, dass sich die Heterogenität der Pharmaziecurriculainhalte in Europa seit Bologna nicht wesentlich verändert hat, aber der Anteil an medizinwissenschaftlichen Inhalten innerhalb der BSc/MSc-Studiengänge gestiegen ist [43], Ergebnisse, die sich ebenso in einer Studie aus dem Jahr 2021 widerspiegeln [44]. Diese Variabilität der Pharmaziecurricula wurde von Atkinson [68] begrüßt, da sie den Akademikern und dem Lehrpersonal die Freiheit gibt, neue Lehr- und Lernansätze in den Studiengängen zu entwickeln. Allerdings hat eine 2020 veröffentlichte globale Vergleichsstudie von Pharmaziecurricula gezeigt, dass sich in Europa viel mehr tun muss, um den Studierenden eine breite Berufswahl im Angesicht der globalen Herausforderungen zu ermöglichen [45]. Hierzu gehören unter anderem praxisgerechte Lernansätze, Integration von Theorie und Praxis sowie didaktische Konzepte, die „Just-in-time“-Lehre fördern. In Ländern, in denen Apotheker zudem als Fachkräfte des öffentlichen Gesundheitswesens angesehen werden, umfassen die Curricula häufig die Aufklärung über das öffentliche Gesundheitswesen und die Verabreichung von Impfungen [46].

Organisationen wie die Centers for Disease Control and Prevention (CDC), Public Health England und die WHO ermutigen kommunale Organisationen und andere Fachleute des Gesundheitswesens, bei der effektiven Planung öffentlicher Gesundheitsdienste mit Apothekern zusammenzuarbeiten [47]. Während der COVID-19-Pandemie beispielsweise übernahmen Apotheker erweiterte Aufgaben bei der Impfung (Australien, Kanada, Deutschland, Irland, die Schweiz, das Vereinigte Königreich und die USA), Patientenaufklärung und Herstellung, um Lieferengpässe zu umgehen, was sich bereits vielerorts in Anpassungen der Curricula niedergeschlagen hat [48]. Dass die sich wandelnde Struktur des Gesundheitswesens ein treibender Faktor ist, zeigt sich auch in den Ländern Asiens, in denen sich weder das Gesundheitssystem noch das Pharmaziestudium einer großen Änderung unterzogen haben. Hier ist immer noch der BPharm-Abschluss vorrangig. Aber in den Ländern Asiens, die eine gesundheitspolitische Weiterentwicklung durchmachen (z. B. Saudi-Arabien, Indien, Pakistan) findet auch seit einiger Zeit eine Weiterentwicklung des Curriculums zum PharmD statt [49].

### Wirtschaftlicher und gesundheitsökonomischer Einfluss auf das Pharmaziestudium

Zusätzlich zu der Anpassung des Gesundheitswesens üben wirtschaftliche und gesundheitsökonomische Aspekte ebenfalls einen erheblichen Einfluss auf die Lehrpläne der Universitäten aus. Die Pharmakoökonomie ist heute ein wichtiger Bestandteil vieler Lehrpläne in der Pharmazie und lehrt die Studierenden, die Kosteneffizienz von Medikamenten und Gesundheitsmaßnahmen zu bewerten [50]. Dies ist von entscheidender Bedeutung, um sicherzustellen, dass Apotheker fundierte Entscheidungen treffen können, die ein Gleichgewicht zwischen einem möglichst guten Ergebnis für die Patienten und den Kosten für die Gesundheitsversorgung herstellen [51].

### Qualitätsstandards und Didaktik in der Lehre

Mit den sich wandelnden Curriculumsanforderungen haben sich auch die didaktischen Ansätze und Qualitätsstandards weiterentwickelt. Verschiedene Berufsverbände, wie der Internationale Pharmazeutische Verband (FIP), haben globale Kompetenzrahmen für das Pharmaziestudium entwickelt [52]. Kompetenzrahmen umreißen die spezifischen Fähigkeiten, Kenntnisse und Verhaltensweisen, die von Apothekern erwartet werden, und dienen als Orientierungshilfe für die universitäre Gestaltung von Pharmaziecurricula. Diese Rahmenwerke werden von Berufsverbänden und Akkreditierungsagenturen entwickelt und spiegeln häufig globale Trends in der Gesundheitsversorgung wider [53]. Auch die Europäische Kommission hat im Mai 2024 ihre neueste Fassung zu den Mindestanforderungen an Berufsausbildung für Apotheker veröffentlicht [54].

Viele Länder weltweit haben ihren eigenen Standard für Pharmaziecurricula, deren Einhaltung von strengen Kontrollen durch Berufsverbände überwacht wird, was erheblich dazu beiträgt, sicherzustellen, dass die Studiengänge auf dem neuesten Stand des wissenschaftlichen Fortschritts sind und den technologischen Innovationen und den Anforderungen des Gesundheitssystems entsprechen [55]. In Europa setzen sich die „European Association of Faculties of Pharmacy“ (EAFP) und die „Pharmaceutical Group of the European Union“ (PGEU) für eine europaweite Angleichung der Pharmaziecurricula und die Harmonisierung der Standards für das Pharmaziestudium ein [56]. Eine Harmonisierung der Standards erlaubt genügend Flexibilität, um die Studienmodelle innerhalb Europas, die vom Bologna BSc/MSc abweichen, beizubehalten (z. B. das Staatsexamen in Deutschland und das „Diplôme d’État de Docteur en Pharmacie“ in Frankreich) und dennoch länderübergreifende Wechsel- und Anerkennungsmöglichkeiten zu ermöglichen [3, 4]. Dies ist besonders vor dem Hintergrund des rasanten Wachstums von Technologien und digitalen Gesundheitsinnovationen sowie der Integration von künstlicher Intelligenz (KI) im Gesundheitswesen wichtig, da die Weiterentwicklung von Pharmaziecurricula und die Einführung von Kompetenzrahmen und Akkreditierungsstandards von entscheidender Bedeutung sind, um sicherzustellen, dass künftige Apotheker auf die Herausforderungen eines sich rasch wandelnden Gesundheitssystems auch in Zeiten des technologischen Fortschritts des 21. Jahrhunderts vorbereitet sind.

### Prinzipien der horizontalen und vertikalen Integration des Pharmaziestudiums

**Abb. 3:**
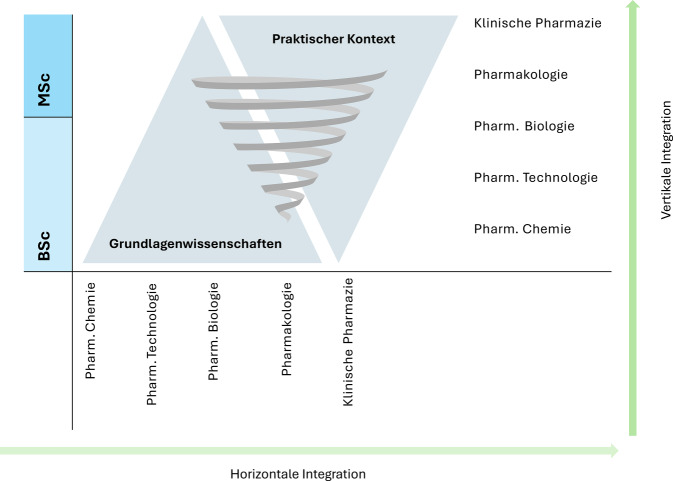
Horizontale und vertikale Integration von Fachgebieten im 5‑jährigen Masterstudiengang für Pharmazie (Bachelor of Science/Master of Science). (Quelle: eigene Abbildung)

Das moderne Pharmaziecurriculum und viele andere universitäre Studiengänge für Gesundheitsberufe versuchen, den Schwerpunkt auf die horizontale und vertikale Integration der Inhalte zu legen [57]. Das bedeutet zum einen die Zusammenarbeit zwischen den Disziplinen, um ein kohärentes Curriculum zu erstellen (horizontale Integration), und zum anderen die Integration zwischen dem praktischen und dem grundlagenwissenschaftlichen und anwendungsbezogenen Teil des Studiums (vertikale Integration). Vertikal integrierte Curricula stellen ab Beginn des Pharmaziestudiums die Zusammenhänge der Grundlagenwissenschaften mit der pharmazeutischen Praxis dar. Bei dieser Art von Lehrplan sind die Grundlagen- und anwendungsbezogenen Wissenschaften von Anfang an miteinander verwoben, sodass die Studierenden die Zusammenhänge schon in einem frühen Stadium ihres Studiums verstehen können [58]. Die vertikale Integration zielt auf die professionelle Entwicklung der Studierenden ab und soll ihnen ermöglichen, sich in einem sich schnell wandelnden Berufsumfeld zurechtzufinden, mit neuen Herausforderungen zurechtzukommen und die Bedeutung der kontinuierlichen beruflichen Weiterbildung zu erkennen (Abb. 3).

In Anbetracht der eindeutigen Vorteile des integrierten Curriculums macht es Sinn, diese Art von Struktur zum universitären Bildungsstandard zu erklären. Dennoch geht sie nicht weit genug. Koens et al. (2005) vermuten, dass das Lernen im Kontext stärker ist als die semantische oder physische Lehre [59] „Ein Curriculum, das nur kognitive Verknüpfungen mit der klinischen Praxis oder die Gelegenheiten zur Beobachtung der klinischen Praxis vorsieht, versäumt es die Studierenden auf eine klinische Verantwortung vorzubereiten“ [60]. Das bedeutet konkret, dass ein vertikal integriertes Pharmaziecurriculum Folgendes beinhalten muss, um wirklich effektiv zu sein [57]:

- die frühe klinische Erfahrung,
- die Integration von biomedizinischen Wissenschaften und klinischen Fällen,
- mehrwöchige interprofessionelle Pharmaziepraktika vor allem in den letzten Studienjahren und
- die Förderung eines zunehmenden Maßes an klinischer Verantwortung.

**Abb. 4:**
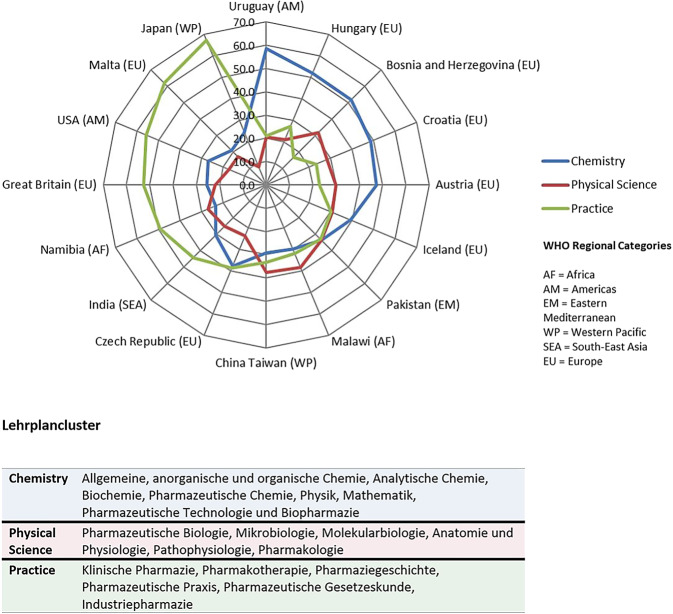
Anteil der themenbezogenen Kontaktstunden im Pharmaziestudium nach Ländern. Aus [45], für die vorliegende Publikation durch die Autorinnen ergänzt um die Beschreibung der Lehrplancluster. Lizenz „Attribution-NonCommercial 4.0 International“ (CC BY-NC 4.0 Deed; [67])

Während ein solches Curriculumdesign vor allem in den angloamerikanischen PharmD- und MPharm-Pharmaziecurricula vorherrscht, besteht gerade in den breiter aufgestellten MSc-Studiengängen innerhalb Europas der dringende Bedarf, sich um eine stärkere Integration von horizontalen und vertikalen Inhalten zu bemühen, wie Vergleichsstudien aus den Jahren 2016 und 2020 eindrücklich zeigen ([45]; Abb. 4). Relativ neu entwickelte MSc-Pharmaziecurricula in den Niederlanden (landesweiter Rahmenplan), Finnland (University of Helsinki), Belgien (KU Leuven) und Österreich (Paracelsus Medizinische Universität, Salzburg) dienen hierfür als „Best-Practice“-Beispiele. Hierbei werden vermehrt moderne didaktische Konzepte wie „Problem-“ und „Team-based Learning“, kompetenzbasierte Lehre, fachübergreifende Prüfungen, Praxissimulationen („The Pharmacy Game“) und interprofessionelle Langzeitpraktika angewendet [61, 62].

### Praktika

Wie oben beschrieben sollten Pharmaziestudierende die Möglichkeit haben, mehrwöchige interprofessionelle Pharmaziepraktika zu absolvieren, die ein zunehmendes Maß an klinischer Verantwortung beinhalten. Diese ist besonders wichtig, um die berufliche Identität von Apothekern zu stärken. Laut Khanna et al. liegt die Vermutung nahe, dass in Ländern mit traditionell naturwissenschaftlich geprägtem Pharmaziestudium Apotheker sich nicht als klinische Fachkräfte identifizieren, sich selbst nicht in dieser Rolle sehen und daher auch keine klinischen Fachkräfte „sein“ können [63]. Diese Identität ist aber von entscheidender Bedeutung, um die Rollenentwicklung des Apothekers im interprofessionellen Team zu entwickeln und sich gemeinsam den gesundheitspolitischen und wirtschaftlichen Herausforderungen des 21. Jahrhunderts zu stellen. Hierbei spielt das Pharmaziecurriculum eine entscheidende Rolle [64].

## Fazit

Weltweit orientieren sich Pharmaziecurricula an den Fortschritten in den pharmazeutischen Wissenschaften und den Bedarfen in der Arzneimittelentwicklung und -versorgung. Dabei hat sich der Tätigkeitsbereich des Apothekers in den letzten Jahrzehnten kontinuierlich erweitert. Ziel muss es sein, Apotheker auszubilden, die als essenzielle Akteure im jeweiligen Gesundheitssystem die sich wandelnden Aufgaben in Anbetracht der zahlreichen globalen Herausforderungen verantwortlich übernehmen können. Die horizontale und vertikale Integration der Studieninhalte tragen maßgeblich zum Erwerb der benötigten Kernkompetenzen bei. Unabhängig vom Bildungssystem besteht die Notwendigkeit einer kontinuierlichen Anpassung der Curricula, um den Ansprüchen und Anforderungen an den modernen Apotheker gerecht zu werden. Hier bildet Deutschland keine Ausnahme.
